# A novel technique for simultaneous determination of drugs using magnetic nanoparticles based dispersive micro-solid-phase extraction in biological fluids and wastewaters

**DOI:** 10.1016/j.mex.2020.100952

**Published:** 2020-06-09

**Authors:** Sabah Shiri, Kamal Alizadeh, Naser Abbasi

**Affiliations:** a Department of Chemistry, Lorestan University, Khorramabad, Iran; b Biotechnology and Medicinal Plants Research Center, Ilam University of Medical Sciences, Ilam, Iran; c Department of Pharmacology, Medical School, Ilam University of Medical Sciences, Ilam, Iran

**Keywords:** Ibuprofen, Nanoparticles, Drugs, Extraction, HPLC

## Abstract

In this study, a novel method was developed to measure acidic and basic drugs in biological and wastewater samples. The method used magnetic nanoparticles based on Vortex-Assisted Dispersive Micro-Solid Phase Extraction (SPE) and then identifying with HPLC-UV. The magnetic nanoparticle (Fe_3_O_4_@SiO_2_@Kit-6@NH_2_) has been used as an efficient adsorbent for the extraction of acidic and basic drugs ibuprofen (IFB), fenoprofen calcium (FPC), methocarbamol (MTC), and clonazepam (CZP). The magnetic nanoparticle was characterized by techniques including SEM, XRD, EDX, and FT-IR. The effect of various parameters in the V-D-μ-SPE method was studied completely through the design of the response surface methodology (RSM) of the Box–Behnken design (BBD) based response method and the utility function. The parameters affecting the extraction efficiency were optimized including sample pH, adsorbent amount, absorption time, the salt concentration in the sample solution, CTAB of concentration, desorption time, and the volume of an eluent. After optimization, the limit of detection and calibration curve in the linear range were obtained 0.062–0.32 μg L^−1^ and 0.1–800 μg L^−1^, respectively. Its linear correlation was *R*^2^> 0.9951. The relative standard deviation (*n* = 5) was between 2.4% and 5.1%. Finally, this method was used to determine target analytes in human serum, urine, and wastewater.

• In this study, for the first time, a novel method for the determination of some drugs from human serum, urine, and wastewater samples.
• The Synthesized Fe_3_O_4_@SiO_2_@Kit-6@NH_2_ NPs based V-D-μ-SPE was characterized by techniques including SEM, XRD, EDX, and FT-IR.
• The effects of various parameters in the V-D-μ-SPE methods were studied through the design of the RSM of BBD.

## Graphical abstract

**Figure:**
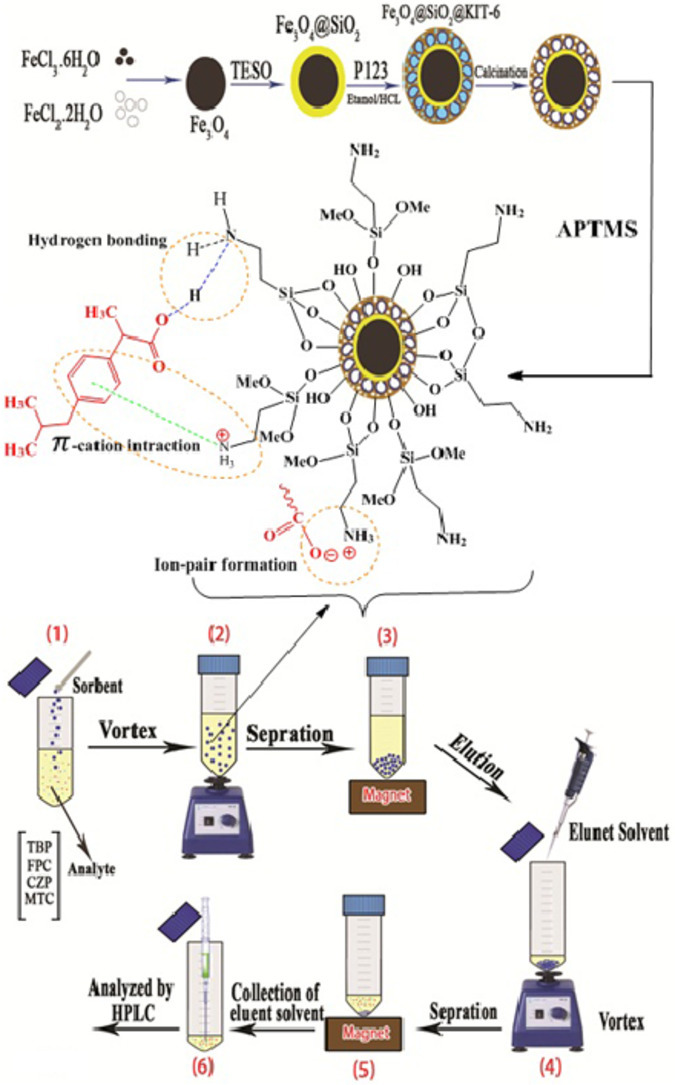

**Specifications table**

**Table:** 

Subject area	More specific subject area	Method name	Name and reference of original method	Resource availability
Human, waste	human serum, urine and wastewater samples	design of the Response Surface Methodology (RSM) of Box-Behnken design (BBD)	Magnetic nanoparticle	Minitab

## Methods details

### Background

**Fig. 1:**
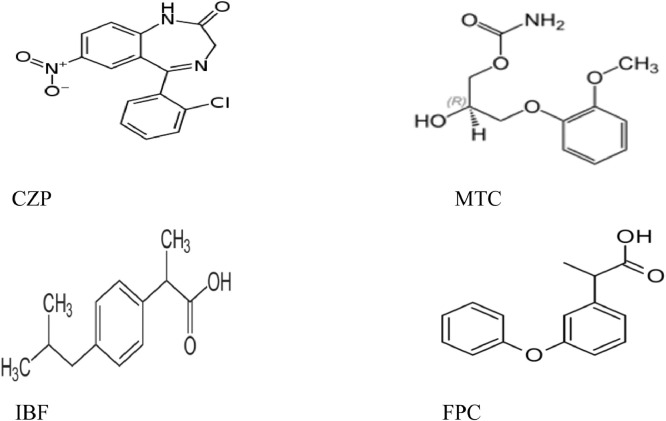
Ibuprofen (IFB), Fenoprofen calcium (FPC), Methocarbamol (MTC), and Clonazepam (CZP) of structures.

In recent years, production and growing worldwide consumption of drugs have become a problem. Accordingly, the identification and measurement of biological and chemical samples are so highly considerable [1,2]. Measurement of amounts of drugs in different samples such as human blood serum and plasma to control the drug level, diagnosis, and assessment of toxicity, is important [3,4]. Measurement of the amounts of some drugs including ibuprofen (IBF), fenoprofen calcium (FPC), methocarbamol (MTC), and clonazepam (CZP) whose molecular structures have been shown in Fig. 1, in vital samples such as human blood serum and plasma to diagnose some diseases, were carried out [5], [6], [7].

Ibuprofen (RS-2(42-methyl propyl) phenyl) propionic acid) is a non-steroidal anti-inflammatory drug (NSAID) with an acid structure that is used as an anti-inflammatory, analgesic, and antipyretic in various diseases [8]. Different forms of this drug enter into the environment through human waste [9]. Also, these forms have been known considerably in underground and surface waters, rivers, non-refined waste, and biological samples [10]. Clonazepam (5-(2-chlorophenyl)7-nitro-2,3-dihydro-1,4-benzodiazepine-2-one) is from the benzodiazepines group which is prescribed as anticonvulsants, sedative, and muscle relaxant [11]. Clonazepam with some main symptoms such as drowsiness and behavioral disorder is in the form of 0.5, 1, and 2 mg tablets [12, 13]. Methocarbamol (R-2--hydroxy-3-(2-methoxyphenyl) propyl carbamate) is used muscular relaxant. Oral and injectable forms can reach a toxic concentration (23.1 ± 2.8 mg/L) in human blood plasma [14]. MTC is absorbed and distributed in intestines easily and extensively and also exists in the whole body tissues especially in the liver, kidney, and blood [15]. Fenoprofen calcium (calcium; 2-(3-phenoxy phenyl) propanoate) derives from propionic acid which is in the category of NSAID [16].

There are different analytical methods such as spectrophotometry [17,18], high performance liquid chromatography (HPLC) [19], [20], [21], [22], [23], [24], gas chromatography (GC) [25], chemiluminescence [26], multi-walled carbon nanotubes nanocomposite modified electrode (voltammetric) [13], high performance liquid chromatography-mass spectrometry (HPLC/MS) [15], fluorescence spectrometry [27], capillary electrophoresis [28,29], supercritical fluid chromatography (SFC) [30], RP-HPLC Method [31,32], square-wave adsorptive anodic stripping voltammetry [7], which mostly have disadvantages such as large sample volume, long time analysis, expensive equipment and toxic solvents [21,[29], [30], [31], [32], [33], [34]]. Therefore developing a simple method, sensitive, fast, selective, and reliable to determine drug compounds in human samples, zoological and the environment is highly important. Due to complex biological samples, low detecting of the device, identification, and determination of the little amount drug in biological samples is required to a preconcentration and extraction method with high performance [35,36]. One of the methods which would be fast, selective, and simple to measure acidic and basic drugs in biological samples and wastewater is D-μ-SPE (Vortex Assisted dispersive micro-solid-phase extraction based magnetic nanoparticles) that is standard to extract some drugs from real samples. It has some advantages such as high preconcentration factors, low consumption of solvent, low extraction time, fast and easy, clean, low sample aqueous phase and green solvents [37]. Micro-solid-phase extraction based magnetic nanoparticles is a new and effective method [38]. So, due to the importance of improving and widespread use of drugs, developing a rapid, sensitive, simple, selective extraction and determination method is essential for the study of biological, clinical, toxic and aquatic samples [39].

One of the well-known quaternary ammonium cationic surfactant is cetyltrimethyl ammonium bromide (CTAB) used to prepare ordered Fe_3_O_4_@SiO_2_@Kit-6@NH_2_ mesoporous silicate molecular sieves at basic conditions [40,41]. CTAB, as a functional monomer, can strongly interact with drugs by electrostatic forces and hydrophobic groups [42,43]. Therefore, the introduction of CTAB functional groups significantly improves the removal efficiency of the adsorbent for the removal of drugs from serum and wastewater.

Optimization of the effective variables on the extraction efficiency of the acidic and basic drugs was conducted by using Mini-Tab software and design express by method response surface methodology (RSM) of Box–Behnken design (BBD), desirable function (DF) and variance analysis ANOVA to evaluate in depended variables effect [44]. The design was determined the effective factors and then a mathematical equation could specify the optimized amount of the tested variables exactly [45]. In this study for the first time, simultaneous determination and extraction of acidic and basic drugs using MNPs (Fe_3_O_4_@SiO_2_@Kit-6@NH_2_) based Vortex assisted dispersive micro-solid-phase extraction from biological fluids and wastewater was carried out.

### Chemicals and reagents

Ibuprofen (IBF), fenoprofen calcium (FPC), methocarbamol (MTC), clonazepam (CZP), ferric chloride hexahydrate, ferrous chloride dehydrate, sodium acetate, aqueous ammonia solution (NH_4_OH), acetonitrile, methanol, ethanol, acetone, cetyltrimethylammonium bromide (CTAB), tetraethylorthosilicate (TEOS), hydrochloric acid, and 3, 5-dinitrosalicylic acid (DNS) were purchased from Merck (Darmstadt, Germany).

Aminopropyltrimethoxysilane (APTMS), 2, 2-Azino-bis (3-ethylbenzothiazoline-6-sulfonic acid) diammonium salt (ABTS), and pluronic P123 (EO_20_–PO_70_–EO_20_) were obtained from Sigma-Aldrich (St. Louis, MO, USA). A stock solution of target analytes (100 mg  L^−1^) was prepared in ethanol and working standard solutions were prepared by dilution of stock solution with double distilled water (DDW).

### Instrumentation

Analysis of IFB, FPC, MTC and CZP using the HPLC system (model platin blue, Knauer, Germany) equipped with a UV- detector (Well chrome, K-2600; Knauer), and a reverse-phase C18 column (length ID 100 × 3 mm, particle size, 3 μm, packing material Eurospher (ІІ) 100–-3 C18, vertex plus column, KNAUER) operating at a wavelength of 256 nm, dual solvent pump (model LC-10Avp) and aerodyne model platin blue injector with 1 μL was done. The mobile phase was made up of acetonitrile and phosphate buffer (30:70, v/v) adjusted to pH=5. The flow rate was set at 0.8 mL  Min^−1^. The pH measurement was done with a 780 pH meter (Metrohm, Switzerland) equipped with a combined Ag/AgCl glass electrode. A Vortex (Biosan model V-1 PLUS, Republic of Latvia) was used in the extraction procedure.

### Preparation of Fe_3_O_4_ @SiO_2_@Kit-6@NH_2_

The synthesis of nanoparticles of Fe_3_O_4_@SiO_2_@Kit-6@NH_2_ was performed in 4 steps: first, 2 gr of FeCl_3_.6H_2_O and 5.46 gr of FeCl_2_.2H_2_O were dissolved in 100 mL of double-distilled water (DDW) under nitrogen atmosphere at 80°C. After that 10 mL of dense ammonia solution added into the previous solution, some black nanoparticles were formed into the solution which throughout the processing time the solution was stirred at 1200 rpm. After 30 min, Fe_3_O_4_ nanoparticles were separated from the solution by using an external magnet and after several times washing by water and to arrive pH=7 were dried by an oven at 50 °c and 24 h [46]. Then, 2 gr of Fe_3_O_4_ nanoparticles were dissolved into 500 mL DDW two times and the mixture was stirred at 30 min by ultrasonic. The magnetic nanoparticles were separated by using a magnet and stirred in a different amount of 2 N ammonia solution (NH_4_OH, 28–30  wt% stock solution (at 80°C and 3 h. The mixture was cooled down at room temperature. In the next step, 3 mL TEOS (98%) which is a silane precursor was dissolved in 100 mL ethanol. This solution with 0.8 mL/min speed dropwise was added to the Fe_3_O_4_ nanoparticles is a suspension solution to cover nanoparticles surface. Finally, brown Fe_3_O_4_@SiO_2_ nanoparticles were separated from the solution by using an external magnet. The separated nanoparticles were then washed with ethanol and distilled water several times and collected [47]. 1.25 gr pluronic P123 was dissolved in 50 mL double-distilled water (DDW) after that 2 gr of Fe_3_O_4_@SiO_2_ and 2.4 mL of concentrated hydrochloric acid solution (37%) were added under mechanical stirring at 1200 rpm. In the next step after 24 h, 2 mL of n-butyl alcohol and 2.4 mL of TEOS were added. The reaction mixture was transferred into a Teflon stainless steel autoclave at 100 °C for 12 h long time was incubated. After the reforming surface, the produced solid material was calcinated by ethanol hydrochloric acid at 550°C at 6 h. The reformed nanoparticles or color brown Fe_3_O_4_@SiO_2_@Kit-6 were separated from the solution by using an external magnet and after several time washing by ethanol and water. The final product dried at 60°C [48]. Surface modified of the core-shell Fe_3_O_4_@SiO_2_@Kit-6 nanoparticles with NH_2_ functionalization was completed by the post-synthesis grafting method using aminopropyl trimethoxysilane (APTMS) in dry toluene. The produced solution was prepared from 1 gr of nanoparticles of Fe_3_O_4_@SiO_2_@Kit-6 in 50 mL of dry toluene at 110°C in next to the 4 mL of aminopropyltrimethoxysilane (APTMS) was refluxed in a round bottom flask at 4 h. The reaction mixture was filtration, and the brown solid was collected by the use of an external magnet and Fe_3_O_4_@SiO_2_@Kit-6@NH_2_ magnetic nanoparticles (MNPs), which was washed with ethanol and DDW and finally dried at 70°C in an oven [49,50].

Magnetic microspheres (Fe_3_O_4_@SiO_2_) with core/shell structures is a desireable support for the preparation of CTAB-functionalized adsorbent. The super paramagnetism of Fe_3_O_4_@SiO_2_ facilitates the rapid separation of the adsorbent by applying a magnetic field from serum and wastewater. On the other hand, the numerous Si–OH groups can ensure a large number of bonded CTAB groups throughout the whole SiO_2_ shell.

### MNPs based D-μ-SPE procedure

**Fig. 2:**
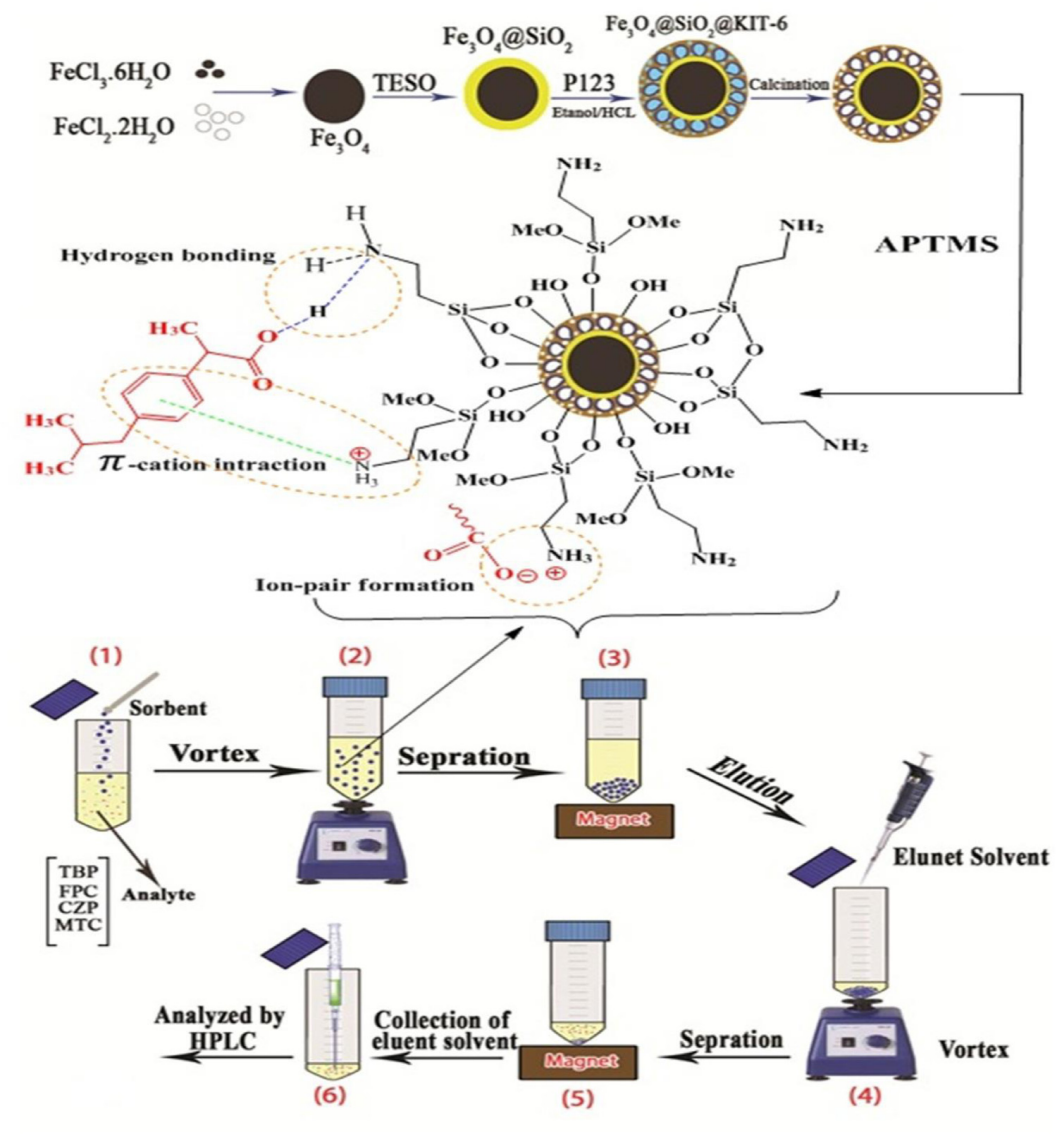
The step of the MNPs based D-μ-SPE method for IBF, FPC, MTC, and CZP.

10 mL of the sample/aqueous standard containing 100 μg. L^−1^ including IBF, FPC, MTC, and CZP, was added and the pH was adjusted to the 5. Subsequently, 25 mg of nanoparticles Fe_3_O_4_@SiO_2_@Kit-6@NH_2_ was added to the sample or standard solution. The mixture was agitated using a vortex for 6 min to facilitate the dispersion of the adsorbent. The magnetic adsorbent was collected by an external magnet and the top solution was decanted and the adsorbent was eluted with 30  μL of methanol. Subsequently, the absorbent mixture and methanol were vortexed at 5 min in other to the complete desorption of the adsorbent. Finally, collecting the absorbent by using a magnet about 1μL of the eluent solvent was injected into the HPLC instrument for subsequent analysis IBF, FPC, MTC, and CZP (Fig. 2).

### Preparation of human serum and urine sample

Human serum and urine samples were collected from healthy volunteers. In other to precipitate proteins and decrease the matrix effect, acetonitrile (3 mL) was added to 1 mL sample. After centrifuging at 2000 rpm for 10 min, the supernatant was collected and diluted with DDW. The urine samples were diluted with DDW and filtered using Whatman No. 42 filter paper and then stored at 4 °C in the dark. Finally, the spike concentrations of IBF, FPC, MTC, and CZP of standard solutions were added into 10 mL prepared blood serum and urine samples to the above-mentioned procedure. A stock solution of target analytes (100 mg L^−1^) was prepared in ethanol and working standard solutions were prepared by dilution of stock solution.

## Analyses

### Characterization of magnetic nanoparticles Fe_3_O_4_@SiO_2_, Fe_3_O_4_@SiO_2_@Kit-6, Fe_3_O_4_@SiO_2_@Kit-6@NH_2_

Sample preparation, in analytical chemistry, is an essential process. One of the most popular sample preparation methods is solid-phase extraction (SPE) [51]. Due to SPE disadvantages such as solvent loss, large secondary wastes, a long procedure, and the need for complex equipment, Dispersive micro-solid phase extraction (DSPE) is developed [52]. DSPE is simple, economic, and easy to perform [38]. Different sorbents including magnetic nanoparticles (MNPs), (such as Fe_3_O_4_@SiO_2_@Kit-6@NH_2_) can be employed with DSPE [53]. They have a significantly higher surface-area-to-volume ratio, a shorter diffusion route, high extraction capacity, rapid extraction dynamics, high extraction efficiencies [39].

**Fig. 3:**
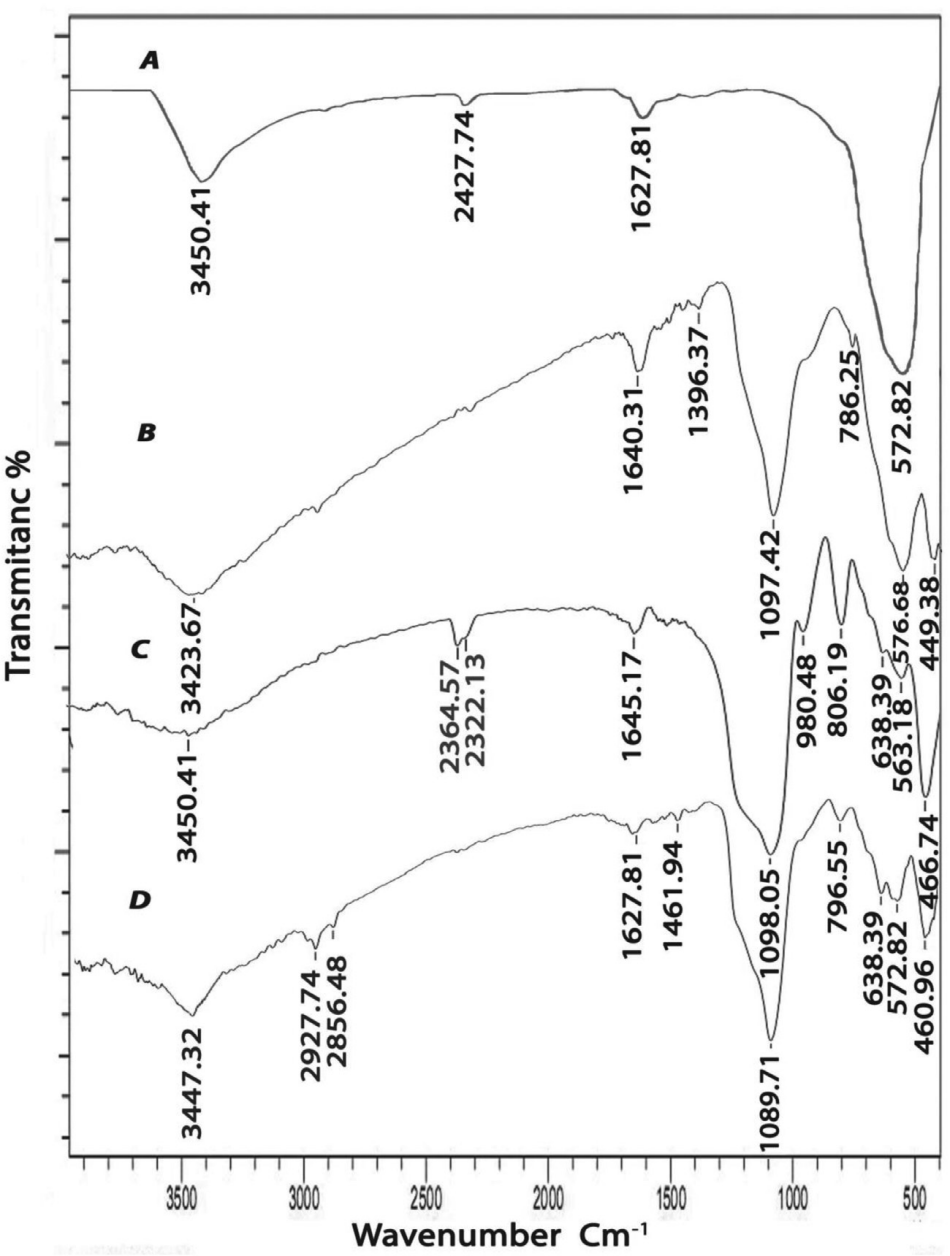
FT-IR spectrum of the prepared Fe_3_O_4_ nanoparticles: in situ TEOS-treated: A) Fe_3_O_4_ B) Fe_3_O_4_@SiO_2_ C) Fe_3_O_4_@SiO_2_@Kit-6 D) Fe_3_O_4_@SiO_2_@Kit-6@NH_2_.

To approve the chemical structure and magnetic property of reformed nanoparticle, some methods such as SEM، EDX, FT-IR, and XRD were used. FT-IR spectra of Fe_3_O_4_, Fe_3_O_4_@SiO_2_, Fe_3_O_4_@SiO_2_@Kit-6, and Fe_3_O_4_@SiO_2_@Kit-6@NH_2_ NPs were examined and the results are shown in Fig. 3. In the FT-IR spectrum of Fe_3_O_4,_ an absorption band appeared at 572.82 cm^−1^ corresponding to the Fe–O bond in the Fe_3_O_4_ particles stretching vibrations and the OH vibration spectrum has been shown at 3450.41 cm^−1^ (Fig. 3A).

In the FT-IR spectrum of Fe_3_O_4_@SiO_2,_ an absorption band appeared at 576.68  cm^−1^ corresponding to the Fe-–O bond (Fig. 3B). The symmetrical stretching vibration, asymmetric stretching vibrations, and binding peaks of siloxane groups (Si–O–Si) were observed wider tapes region at 1097.42, 786.25, and 449.38  cm^−1^, respectively. The peak at 3423.67 cm^–1^ corresponds to the vibration of hydroxyl groups (OH^‐^). This finding is similar in line with those of other studies [39]. In the FTIR spectrum of Fe_3_O_4_@SiO_2_@Kit-6 (Fig. 3C), the Fe-O stretching vibration at 563.18  cm^−1^, the symmetrical stretching vibration peaks of (Si–O–Si) were observed at 980.48 and 806.19  cm^–1^. Bands of C—H symmetrical and asymmetric stretching of propyl chains at 2853.72 and 2922.34  cm^−1^ and bending stretching vibration of O—H at 3450.41 cm^−1^. In the FTIR spectrum of Fe_3_O_4_@SiO_2_@Kit-6-NH_2_ (Fig. 3D), the characteristic peak presented at 572.82  cm^−1^ verifies the Fe–O vibration, Si–O–Si the symmetrical stretching, asymmetric stretching and binding vibration peaks at 1089.71, 796.55 and 460.96 cm^−1^ (that shown some shifts). Bands of C—H stretching vibrations of propyl chains at 2856.48 and 2927.74 cm ^−1^ and stretching vibration of N—H of amine functional groups were observed at 3447.32  cm^−1^.

**Fig. 4:**
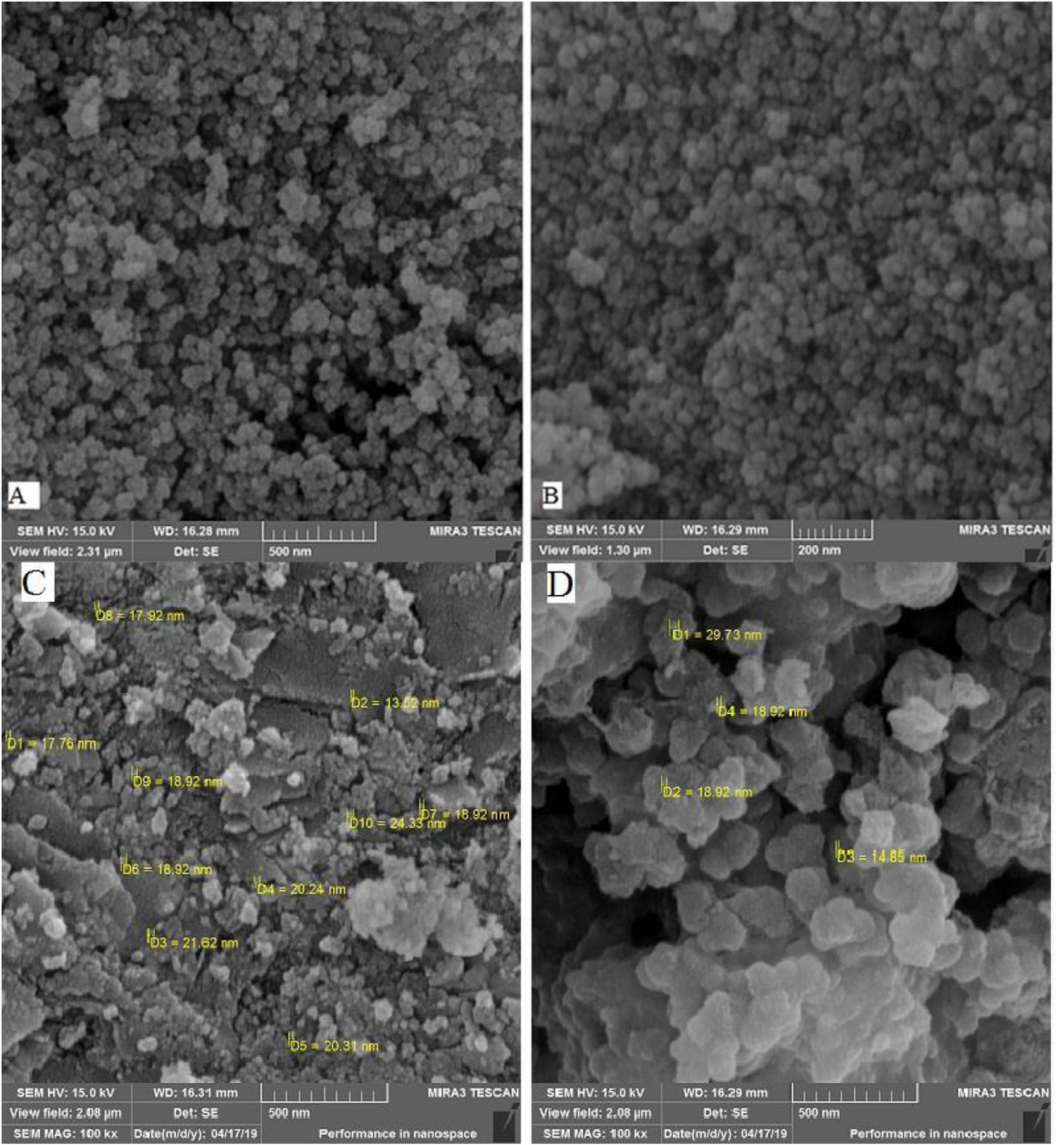
SEM A) Fe_3_O_4_ B) Fe_3_O_4_@SiO_2_ C) Fe_3_O_4_@SiO_2_@Kit-6 D) Fe_3_O_4_@SiO_2_@Kit-6@NH_2_ spectrum nanoparticles.

Scanning electron microscopy (SEM) (TESCAN, model FESEM, Czech Republic) was *used* to characterize the *surface morphology*, size estimate, and shell nucleus structure of obtained nanoparticles (Fig. 4). The SEM showed that nanoparticles shape is about spherical (Fig. 4 A, B). SEM of the reformed nanoparticles shows a considerable cavity structure and surfaced increasing of nanoparticles (Fig. 4C, D). It has been shown that the mesoporous shell was expended on Fe dark magnetic nuclei surface [19,20]. As shown in (Fig. 4C and D), Fe_3_O_4_@SiO_2_@Kit-6 NPs and Fe_3_O_4_@SiO_2_@Kit-6@NH_2_ NPs are nearly spherical with an average diameter of 13.52–24.33 and 14.85–29.72 nm, respectively that is following previous studies [54]. From obtained results it is clear that ultrafine spherical NPs with mean diameter of about 20 nm are almost uniformly dispersed over the surface of mesoporous KIT-6 through – (CH_2_)_3_–NH_2_. A dark Fe_3_O_4_ magnetic core is also easily recognizable from its position and larger size.

**Fig. 5:**
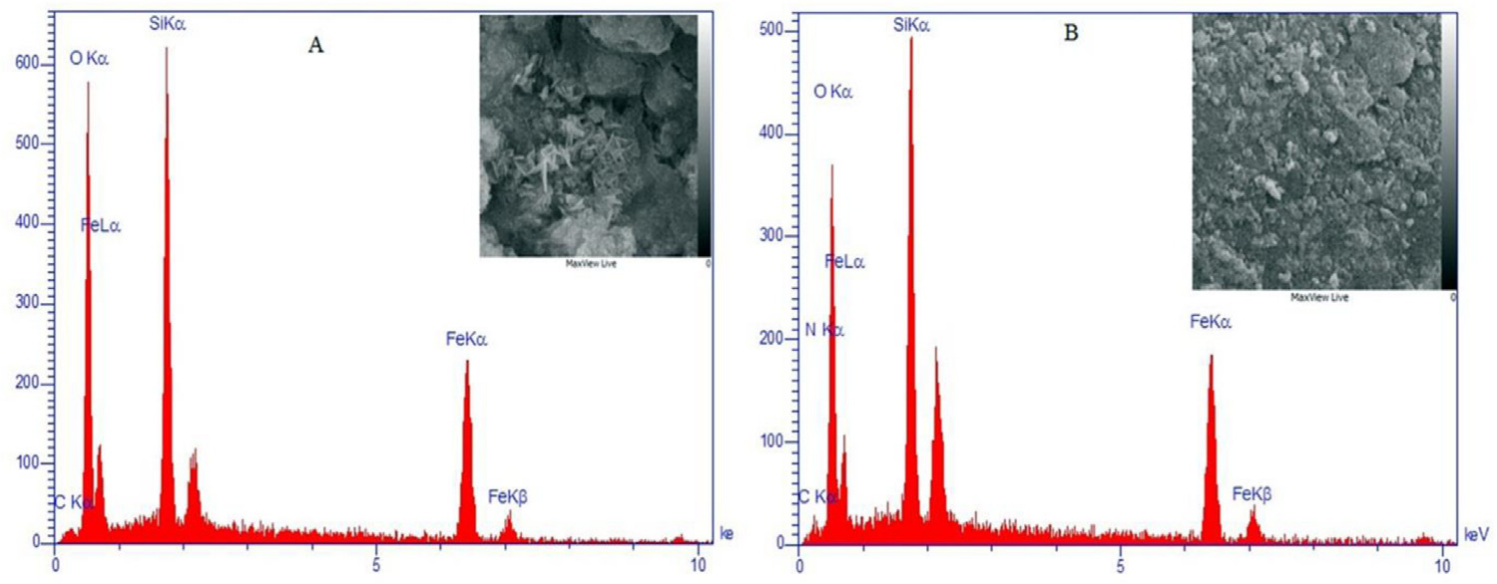
Diffraction spectrum of energy-dispersive X-ray EDX for nanoparticles A: Fe_3_O_4_@SiO_2_@Kit-6 and B: Fe_3_O_4_@SiO_2_@Kit-6@NH_2_.

Energy dispersive X-ray spectroscopy (EDX) is an analytical way to structural analysis and discovery or chemical feature of a sample was used (Fig. 5). Fig. 5A showed some elements such as Fe, C, Si, and O referred to be a reformed nanoparticle surface with a pluronic P123 compound. Fig. 5B also showed all the mentioned elements and nitrogen that indicate the NH_2_ agent group at the nanoparticle structure is final. It previously studied the behavior of Fe3O4 vibrating sample magnetometer, before and after mixing with other materials, and the results indicated similar behavior of hysteresis but with lower saturation magnetization by its content in the mixture [55].

**Fig. 6:**
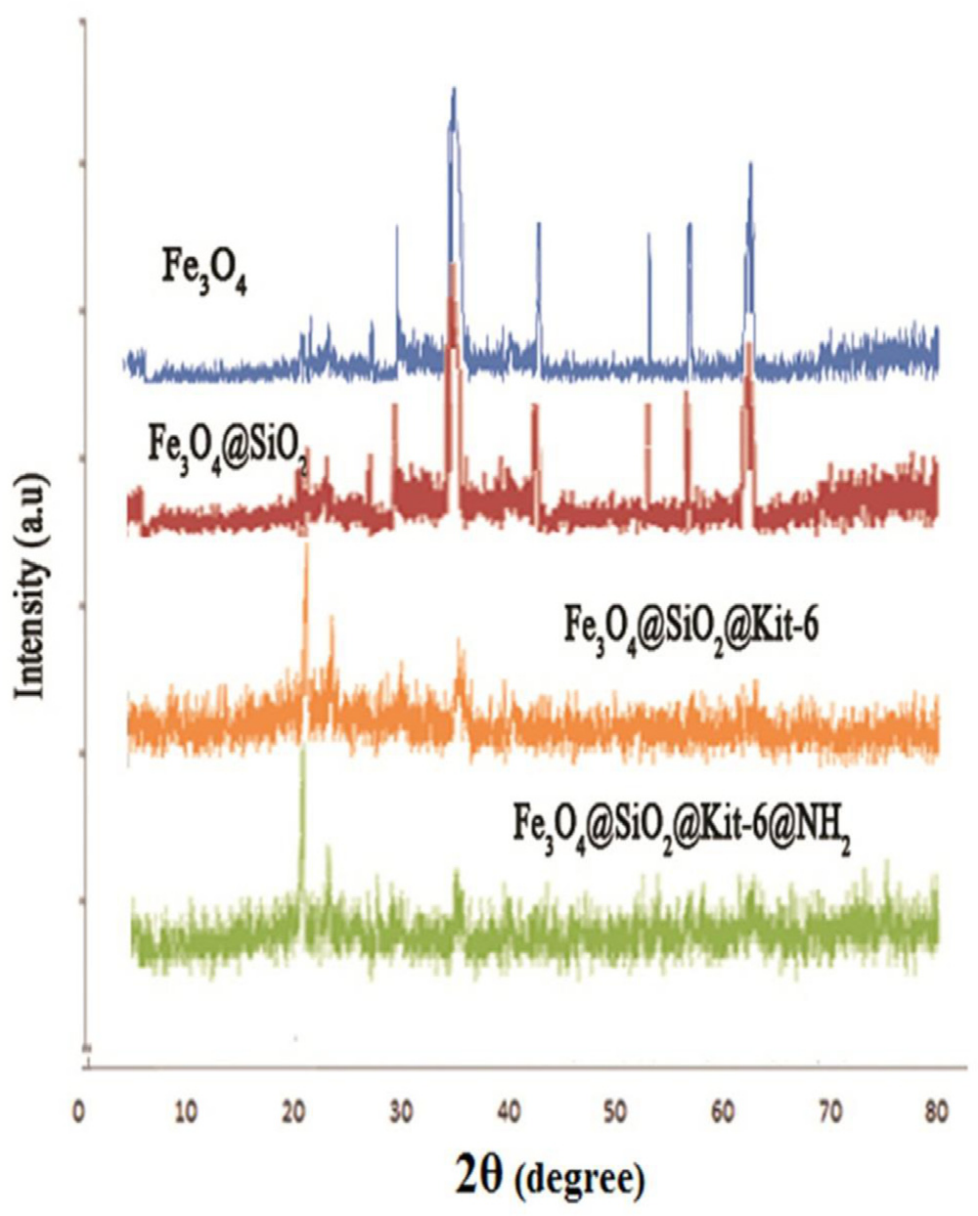
XRD pattern Fe_3_O_4_, Fe_3_O_4_@SiO_2_, Fe_3_O_4_@SiO_2_@Kit-6, and Fe_3_O_4_@SiO_2_@Kit-6@NH_2_.

For the analysis of nanostructures, XRD has good potential because of the width and shape of reflections yield information about the substructure of the materials [51]. The XRD patterns of Fe_3_O_4_ and Fe_3_O_4_@SiO_2_ were presented in Fig. 6. Six peaks at 30.3°, 35.6°, 43.1°, 53.2°, 57.6°, and 62.7 ° with 2θ in the XRD spectra were related to hexagonal shape in Fe_3_O_4_. For Fe_3_O_4_@SiO_2_@Kit-6, three peaks appeared 21.44°, 35.78°, and 62.90° that related to the small particle size for adsorbed kit-6 on the surface. Finally, for Fe_3_O_4_@SiO_2_@Kit-6@NH_2_, two peaks were observed at 21.44°, 35.78° that showed surface coating with amino functional groups.

### Optimization of D-µ-SPE extraction conditions

**Fig. 7:**
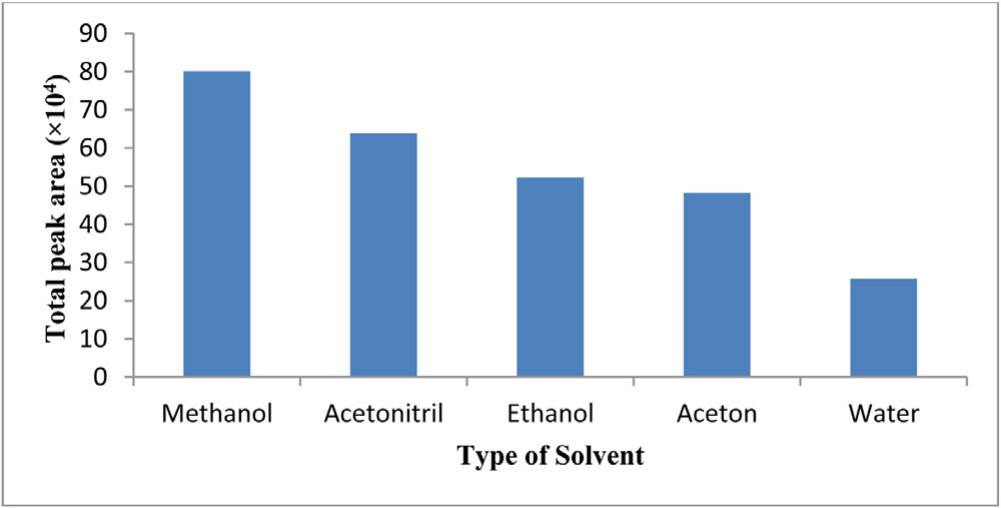
Effects of the eluent type on the extraction efficiency of IBF, FPC, MTC, and CZP. The same extraction conditions.

The choice of a suitable elution solvent is very important to complete the desorption of species from the adsorbent surface related to polarity, species solubility, green solvent, availability, and compatibility. Some different solvent such as acetonitrile, ethanol, methanol, and water were examined (Fig. 7). According to the results, the number of desorption samples from an adsorbent surface using methanol increased in comparison to some other solvent. The extraction performance decreased with arising elution solvent volume.

### Experimental design methodology and desirability function Plackett–Burman designs (PBD)

**Fig. 8:**
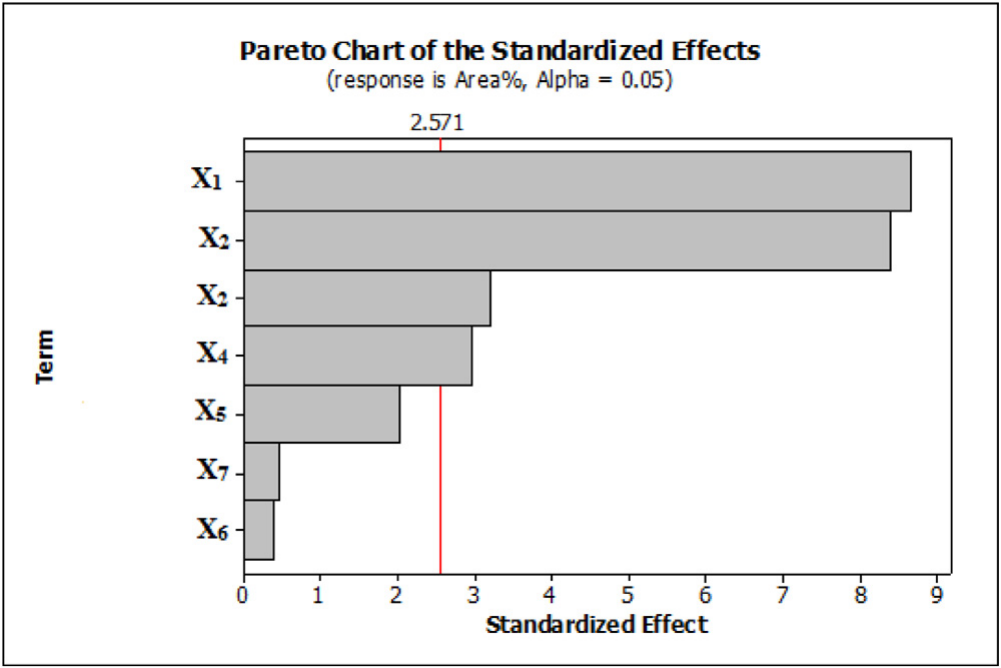
Pareto chart for the PB. The vertical line in the chart defines the 95% confidence level and extraction of IBF, FPC, MTC, and CZP using D-μ-SPE method.

Plackett–Burman design is one of the strongest tools in quick searching of key variables and important at multivariable systems. So, it is one of the most useful methods in the first stages of optimization [26]. The affective variables on the extraction performance were screened by PBD and optimized by BBD. The factors affecting the extraction efficiency of the proposed method such as the amount of adsorbent, pH, Volume of eluent solvent, absorption time, desorption time, NaCl concentration, and CTAB concentration (mM) were investigated and optimized. It has been reported the variable surface, the coded and actual values of factors, the number of essential experiments, and respective responses for each test (Table 1). The experiments were accomplished randomly decrease to non-controlled variables effect. In this plan, two central points were used to estimate the error and repeatability of the system. The obtained responses were analyzed using a Pareto chart (Fig. 8). It indicated that some variables affecting experiment response, include the amount of adsorbent (mg), the volume of eluent solvent (μL), pH, and absorption time (min) which crosses significant factors with a 95% confidence level. Three other variables, including ionic strength, desorption time, and CTAB concentration do not have a significant effect on experiment response. However, it has been demonstrated that CTAB has a prominent role in the extraction mechanism of the drugs. It was found that in optimized pH by increasing the amount of CTAB, the adsorption efficiency will increase. On the other hand, at high concentrations of CTAB, the adsorption efficiency decreased due to the formation of CTAB micelles [56].

**Table 1:** Factors, actual and codded levels, and design matrix used in PBD for the extraction of IBF, FPC, MTC, and CZP using D-μ-SPE method.

	Levels							
Factors	Low (−1)	Central (0)	High (+1)					
(*X*_1_) amount of adsorbent (mg)	5	17.5	30					
(*X*_2_) pH	2	7	12					
(*X*_3_) volume of eluent solvent (μL)	30	65	100					
(*X*_4_) absorption time(min)	4	7	10					
(*X*_5_) desorption time (min)	5	10	15					
(*X*_6_) NaCl concentration (%w /V)	0	5	10					
(*X*_7_) CTAB concentration (mM)	0	15/0	3/0					

Run	*X*_1_	*X*_2_	*X*_3_	*X*_4_	*X*_5_	*X*_6_	*X*_7_	Total peak area(× 10^4^)

1	5.0	30	12	5	15	0	0.30	28.5
2	30.0	100	12	5	5	10	0.30	24.3
3	5.0	100	2	15	5	10	0.00	20.3
4	5.0	30	2	15	15	10	0.30	41.8
5	30.0	100	2	15	15	0	0.30	45.1
6	5.0	100	12	5	15	10	0.00	18.2
7-Cp	17.5	65	7	10	10	5	0.15	75.9
8-Cp	17.5	65	7	10	10	5	0.15	72.4
9	5.0	100	12	15	5	0	0.30	15.4
10	5.0	30	2	5	5	0	0.00	33.5
11	30.0	100	2	5	15	0	0.00	35.9
12	30.0	30	12	15	15	10	0.00	50.9
13	30.0	30	12	15	5	0	0.00	52.4
14	30.0	30	2	5	5	10	0.30	50.7

Cp– Centre Point

### Box– Benken designs (BBD)

It is one of the most practical optimization methods which would prepare possible variables coefficients searching at a mathematical equation and variables optimal conditions forecasting and also response evaluation. The Box–Behnken design expressed in Eq. (1) is a second-order polynomial model based on incomplete factorial design and has some wide usage in determining testing optimal conditions [28]. Each one of the dependent variables in three levels was defined based on coded values low level (−1), medium level (0), and high level (+1) [28], [29], [30], [31].

(1) $$Y=\beta_{0}+\sum_{i=1}^{4}\beta_{i}X_{i}+\sum_{i=1}^{4}\beta_{ii}{X_{i}}^{2}+\sum_{i,j=1\left(i\neq j\right)}^{4}\beta_{ij}X_{i}X_{j}+\varepsilon$$

In this equation *Y* is the analytical response (total peak area), *β*_0_ is model constant, *β_i_, β_ii_, β_ij_* are linear, squared and interaction coefficients, respectively. The number of experimental points (*N*) is defined as follows:

$$N=2K\left(K-1\right)+C_{P}$$

Where that *N* is the test numbers, *K* is the variables numbers and *Cp* is the center point's numbers. Center points were done to obtain an estimation of experimental errors.

Table 2 summarizes coded and actual values, the experimental design, and response values for the extraction of target analytes. To decrease the effect of the uncontrollable variable, the experiments were accomplished randomly. After choosing a suitable mathematical model, the statistical data analysis, searching for suitability of the laboratory model, and also the calibration curve was done using design expert and Mini-Tab Software. After identifying the significant variables in PB method, the relationship between three independent factors (the amount of adsorbent (mg), the volume of eluent solvent (μL), pH and absorption time (min)) for the extraction of IBF, FPC, MTC, and CZP were investigated based on BBD. To prevent some uncontrollable errors, BBD tests were accomplished randomly. The obtained responses of Table 2 were analyzed using the Pareto chart (Fig. 8).

**Table 2:** Factors, actual and codded levels, and design matrix used in BBD for the extraction of IBF, FPC, MTC, and CZP using D-μ-SPE method.

Factors			Levels			
			High (+1)	Central (0)	Low (−1)	
(*X*_1_) amount of adsorbent (mg)			30	17.5	5	
(*X*_2_) pH			12	7	2	
(*X*_3_) eluent solvent (μL)			100	65	30	
(*X*_4_) absorption time (min)			10	7	4	

Run	(*X*_1_)	(*X*_2_)	(*X*_3_)	(*X*_4_)	Total peak area(× 10^5^) (actual value)	Predicted value (× 10^5^)
1	17.5	100	2	7	42.3	39.37

2	17.5	30	7	10	68.4	65.45
3	5	100	7	7	29.2	23.35
4-Cp	17.5	65	7	7	66.4	62.93
5	17.5	65	12	10	31.2	29.08
6	30	65	7	4	70.1	66.05
7	30	30	7	7	80.2	86.35
8	17.5	30	2	7	58.4	56.12
9	5	65	7	4	24.3	26.25
10	17.5	65	2	4	41.5	43.92
11	30	65	7	10	53.7	52.25
12	5	65	7	10	23.4	27.95
13	30	100	7	7	35.3	37.70
14	30	65	2	7	58.2	58.22
15	17.5	100	12	7	13.6	16.38
16	17.5	65	12	4	43.3	43.08
17	5	30	7	7	38.7	36.60
18-Cp	17.5	65	7	7	64.3	62.93
19	17.5	100	7	4	38.4	40.55
20	17.5	65	2	10	45.3	45.82
21	5	65	12	7	18.2	17.38
22-Cp	17.5	65	7	7	58.1	62.93
23	5	65	2	7	15.6	17.87
24	17.5	100	7	10	32.7	34.15
25	30	65	12	7	44.2	41.13
26	17.5	30	12	7	58.1	61.53
27	17.5	30	7	4	73.4	71.15

Cp – Center point

Analysis of Variance (ANOVA) was used to perform the obtained responses' significance. *P*-values less than 0.05 (*p*-value <0.05) are shown that experimental data is suitable. In this study, *P*-value was 0.5662 and the determination coefficient of the design model was 0.9733.

The quadratic model below would show the relationship between the analytical response of total peaks area (*Y*) and significant variables.

$$\begin{array}{ccc} Y & = & -98.77\times 10^{6}+13.72\times 10^{6}X_{2}+6.68\times 10^{5}X_{1}-5.92\times 10^{4}X_{3}-12.87\times 10^{6}X_{4}-8.5\times 10^{3}X_{1}^{2}\\ & & -2.95\times 10^{2}X_{3}^{2}-6.4\times 10^{4}X_{2}^{2}-7.2\times 10^{4}X_{4}^{2}-6.64\times 10^{3}X_{1}X_{2}-4.06\times 10^{3}X_{2}X_{3}-2.022\times 10^{3}X_{1}X_{3}\\ & & -1.04\times 10^{4}X_{1}X_{4}-2.65\times 10^{4}X_{2}X_{4}-1.67\times 10^{2}X_{3}X_{4} \end{array}$$

Where *Y* is the area of the total peak (*Y*) factor of analytes, *X*_1_ is the amount of adsorbent, *X*_2_ is the volume of eluent solvent, *X*_3_ is pH and *X*_4_ is absorption time.

**Fig. 9:**
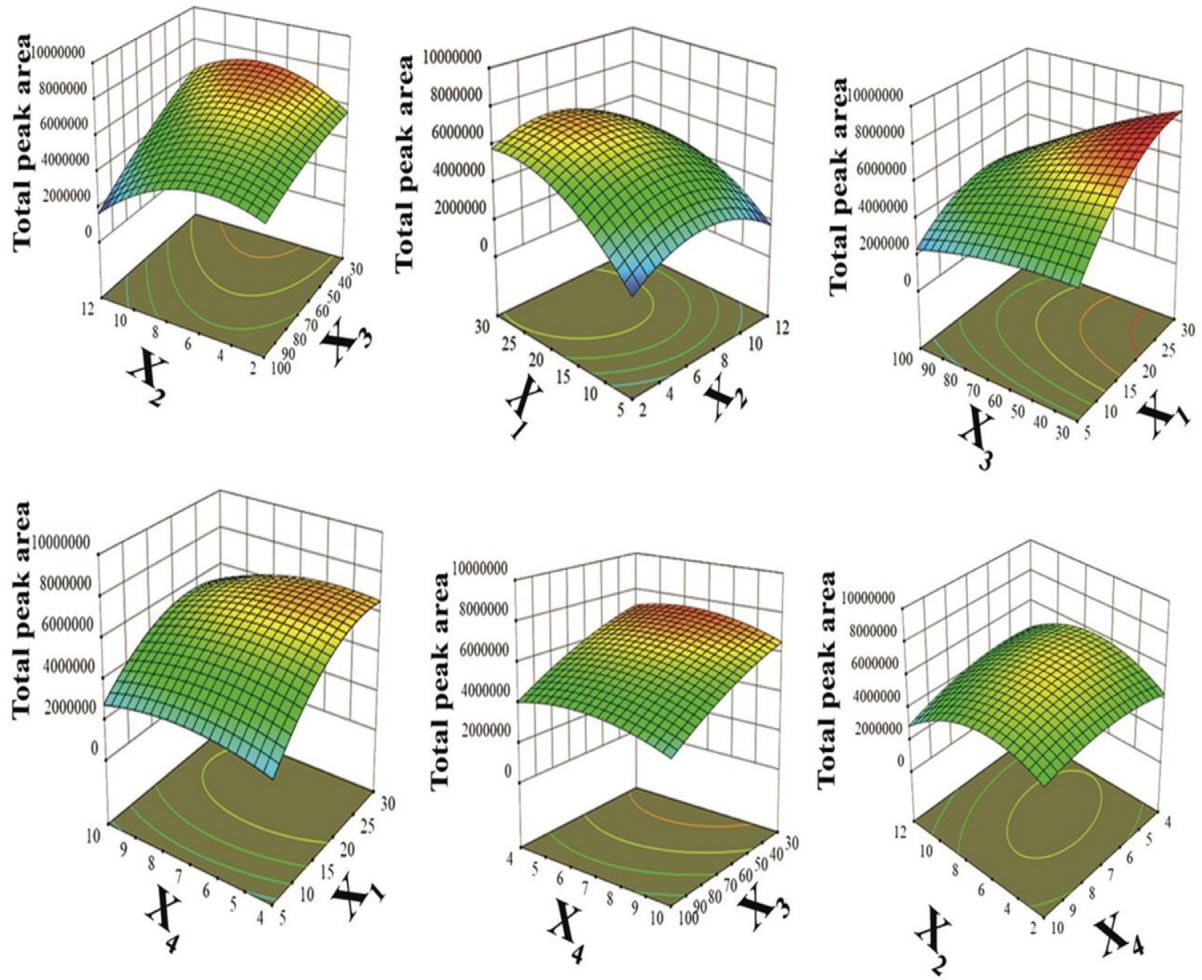
3D plots of significant factors: (*X*_1_: the amount of adsorbent (mg); *X*_2_: pH *X*_3_: volume of eluent solvent (μL) and *X*_4_: absorption time (min) using BD to the extraction of IBF, FPC, MTC and CZP using D-μ-SPE method.

Fig. 9 showed the various 3D plots and interactions between variables. The results showed that by increasing the adsorbent value from 5 to 30 mg, the extraction efficiency had increased. In value less than 25 mg, because of the inadequacy of absorption available surface area for species the extraction efficiency was low. In value more than 25 mg because of nanoparticle collection, the distribution of them in sample aqueous was not done well and the extraction efficiency would decrease.

The effect of eluent volume was investigated in the range of 30–100  μL. The obtained results were indicative of the increasing of analytical response up to 30  μL. The decrease of extraction efficiency by an increasing volume of 30  μL is the dilution of the mentioned species. Volume less than 30  μL, because of volume decreasing and different errors and, the volume analytical error is not suitable. Therefore, this factor was set at 30  μL.

In Fig. 9, the analytical response changes have been represented by the despondent time change. The extraction efficiency has increased by adsorption time (extraction time). In this regard, the maximum response was observed for 6 minutes and more than that, it became a constant and steady-state. By increasing pH to reach neutralizing range, the extraction efficiency has increased and by increasing pH to the basic range, the analytical response decreased, and also most of the analytical response was set at pH= 5.

**Fig. 10:**
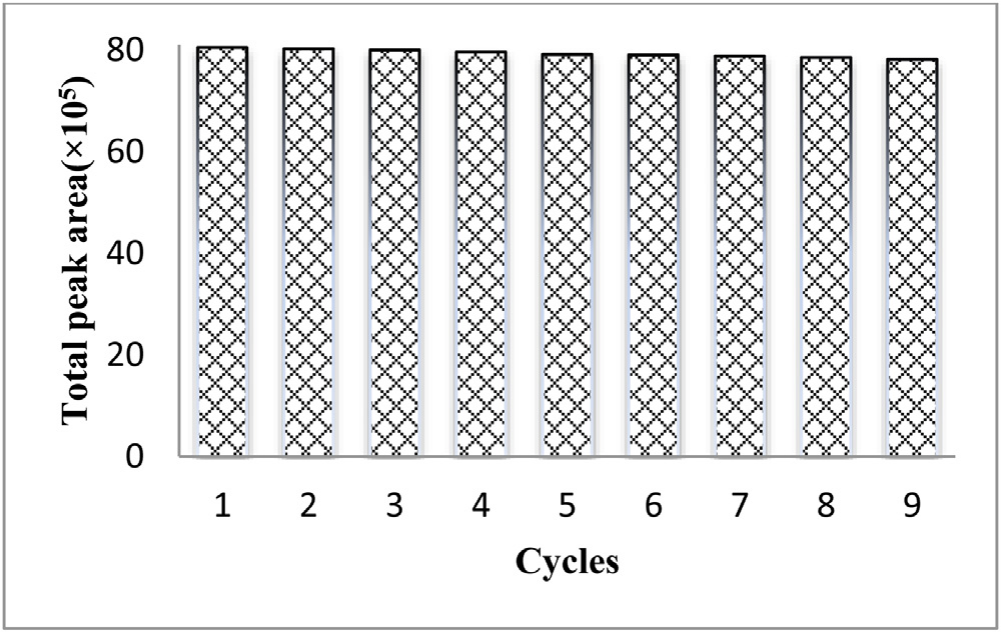
Investigation the *repeatability* of Fe_3_O_4_@SiO_2_@Kit-6@NH_2_ NPs adsorbent.

As shown in Fig. 10, 25 mg of adsorbent, 30  μL of eluent solvent, pH=5 and 6 min of absorption time was chosen as the optimum conditions for the extraction of IBF, FPC, MTC and CZP using D-μ-SPE method.

To investigate the codependency between the obtained optimum predicted value and experimental results, five sets of experiments were accomplished. The results showed a good agreement between the predicted values by the model and the experimental values at the points of interest. The obtained recovery values showed the relative standard deviation (RSD) of analytes replicate extraction from the predicted values which were less than 4.3%. Also, the linear relationship between the predicted values and the experimental values is observable. *R*-squared (*R*^2^) was 98.31 that suggested more than 98% extraction efficiency belongs to the user dependent variables, and just less than 2 percentage of the changes are not justifiable with the experimental model.

### Fe_3_O_4_@SiO_2_@Kit-6@NH_2_ NPs repeatability

To investigate the of Fe_3_O_4_@SiO_2_@Kit-6@NH_2_ NPs adsorbent repeatability after just one-time extraction, the adsorbent was collected by an external magnetic force, and it was used in extraction processes after washing with methanol. The results indicated the nanoparticles efficiency did not decrease by nine times (Fig. 10).

### The capacity of the (Fe_3_O_4_@SiO_2_@Kit-6@NH_2_) NPs adsorption

To study the adsorption capacity of the (Fe_3_O_4_@SiO_2_@Kit-6@NH_2_) NPs, 200 μg/L of IBF, FPC, MTC, and CZP were used as the standard solution. Using the following equation the adsorption capacity for special species was calculated.

$$Q=\frac{\left(C^{\circ }-C\right)\;\times \;V}{g}$$

In this equation *Q, C*°, *C, V, g* is adsorption capacity, initial concentration, extraction after concentration, aqueous sample volume, and adsorbent amount, respectively. According to the equation, the Sorption capacity was found 70.36, 92.68, 45.75, and 73.54 mg/g for MTC, FPC, IBF, and CZP, respectively.

### The study of adsorbent Fe_3_O_4_@SiO_2_@Kit-6@NH_2_ NPs selectivity

The selectivity of modified Fe_3_O_4_@SiO_2_@Kit-6@NH_2_ NPs for the determination of target analytes was studied under optimized conditions using the V-D-μ-SPE method. Ascorbic acid and aspirin may reduce the efficiency of IBF, FPC, MTC, and CZP extraction over time by adsorption on the adsorbent's surface. To evaluate the selectivity of aqueous samples, different concentrations of IBF, FPC, MTC, and CZP in ascorbic acid and aspirin in the concentration range of 0:1 to 10:1 were selected.

**Fig. 11:**
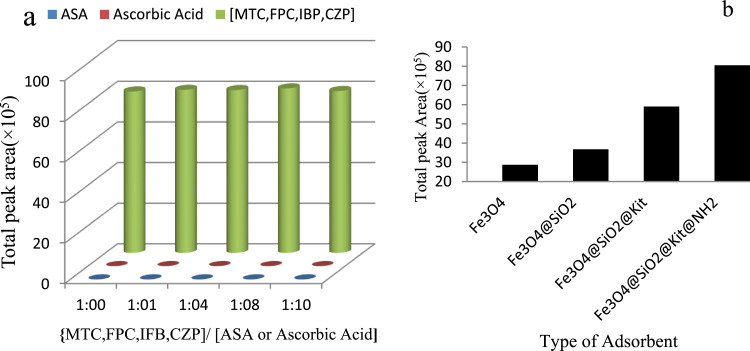
The effect of interference of aspirin) acetylsalicylic acid (ASA) (and ascorbic acid on the extraction efficiency of target analytes (a); comparison the extraction efficiency of modified Fe_3_O_4_@SiO_2_@Kit-6@NH_2_ NPs with Fe_3_O_4_@SiO_2_@Kit-6*,* Fe_3_O_4_@SiO_2_*_,_* Fe_3_O_4_ NPs (b).

Our results indicated that presented ascorbic acid and aspirin would not change the extraction efficiency value (Fig. 11a). More polarity and functional groups of ascorbic acid and aspirin are likely to increase their dissolution in the aqueous phase compared to aromatic structures such as IBF, FPC, MTC, and CZP. Also, the increase in interaction between nanoparticles and IBF, FPC, MTC, and CZP is another reason for the increase in surface absorption and thus the increase in selectivity [17].

The extraction efficiency of Fe_3_O_4_@SiO_2_@Kit-6@NH_2_ for the adsorption of target analytes was compared with Fe_3_O_4_*,* Fe_3_O_4_@SiO_2_*, and* Fe_3_O_4_@SiO_2_@Kit-6 NPs. The results in (Fig. 11b) showed when Fe_3_O_4_@SiO_2_@Kit-6@NH_2_ was used as an adsorbent, the extraction efficiency would increase. This difference in the extraction efficiency may be due to adsorbent surfaced increasing, superficial cavity, the amine groups of MNPs, and the functional groups' target analytes such as hydroxyls, ketone, amine, electron pairs. In the case of Fe_3_O_4_@SiO_2_@Kit-6@NH_2_ NPs, due to the presence of carboxylic groups of drugs, the cloud of electrons of an aromatic ring is lower than that of drugs, resulting in a smaller π–π interaction, ion-pair formation and hydrogen banding of target analytes with the adsorbent.

### Analytical figures of merit of the proposed MNPs based V-D-μ-SPE

To evaluate the proposed method in this study, the figures of merit under the final optimized conditions including linear dynamic ranges (LDRs), limits of detection (LODs), limits of quantification (LOQs), correlation of determination (r2) and extraction recoveries (ER %) were obtained. LODs and LOQs were calculated as 3 s/m and 10 s/m, respectively. Repeatability (within-day RSDs, *n* = 5 samples, at 100 μg  L^−1^ level of the analytes) and reproducibility (between day RSDs, *n* = 3 days, at 100 μg  L^−1^ level of the analytes) of the V-D-μ-SPE method for the determination of the target analytes were less than 3.8 % and 5.1 %, respectively (Table 3).

**Table 3:** Analytical figures of merit of the proposed MNPs based V- D-μ-SPE method for determination and extraction of IBF, FPC, MTC, and CZP.

Analyte	LDR (μg L^−1^)^a^	LOD (μg L^−1^)	LOQ (μg L^−1^)	*r*^2^	RSD% (within a day, *n* = 5)	RSD% (between day, *n* = 3)
MTC	1–800	0.32	1.08	0.9976	3.2	4.8
FPC	0.1–500	0.075	0.25	0.9998	2.4	3.6
IBF	1–600	0.27	0.9	0.9951	3.8	5.1
CZP	0.1–600	0.062	0.21	0.9996	2.9	3.2

a Concentration in μg L^−1^.

### The analysis of blood serum and urine

The evaluation of IBF, FPC, MTC, and CZP using the D-μ-SPE method by adding some different concentrations of the standard solution (*n* = 3) of target analytes into the human serum, urine, and wastewater real samples were done. Serum, urine and wastewater samples were spiked with different concentration levels (10,100 and 300  μg L^−1^) of drugs. ER, % of drugs (IBF, FPC, MTC, and CZP) were reported in three concentration levels of 10,100 and 300  μg L^−1^ for different real samples. As shown in Table 4, RSDs were less than 4.9% while the recoveries were more than 94.3%.

**Table 4:** Determination of IBF, FPC, MTC, and CZP in biological and wastewater samples.

sample	Compound	Amount found (μg L^−1^ ± SD^a^)	Recoveries (RSD %) Amount add (μg L^−1^)		
			10	100	300
Human serum	MTC	15.8	98.7(3.3)	101.3(3.6)	102.5(3.1)
	FPC	ND	96.9(2.5)	97.1(4.5)	95.4(3.8)
	IBF	26.55	99.3(4.1)	100.6(3.2)	103.7(2.2)
	CZP	ND	98.7(2.8)	96.8(3.6)	94.8(2.7)
Urine	MTC	19.2	103.3(3.7)	100.7(4.1)	98.6(2.8)
	FPC	ND	94.8(3.0)	95.3(3.1)	97.5(5.1)
	IBF	32.68	103.6(4.2)	104(3.8)	105.2(5.0)
	CZP	ND	95.9(2.1)	97.2(3.4)	100.7(4.6)
Wastewater	MTC	ND	97.3(3.7)	102.3(2.9)	94.4(3.5)
	FPC	ND	97.4(2.9)	96.8(3.3)	98.9(3.7)
	IBF	ND	94.3(2.3)	98.9(4.2)	99.2(2.6)
	CZP	ND	98.4(2.9)	94.6(3.7)	99.6(4.6)

a Standard deviation.

**Fig. 12:**
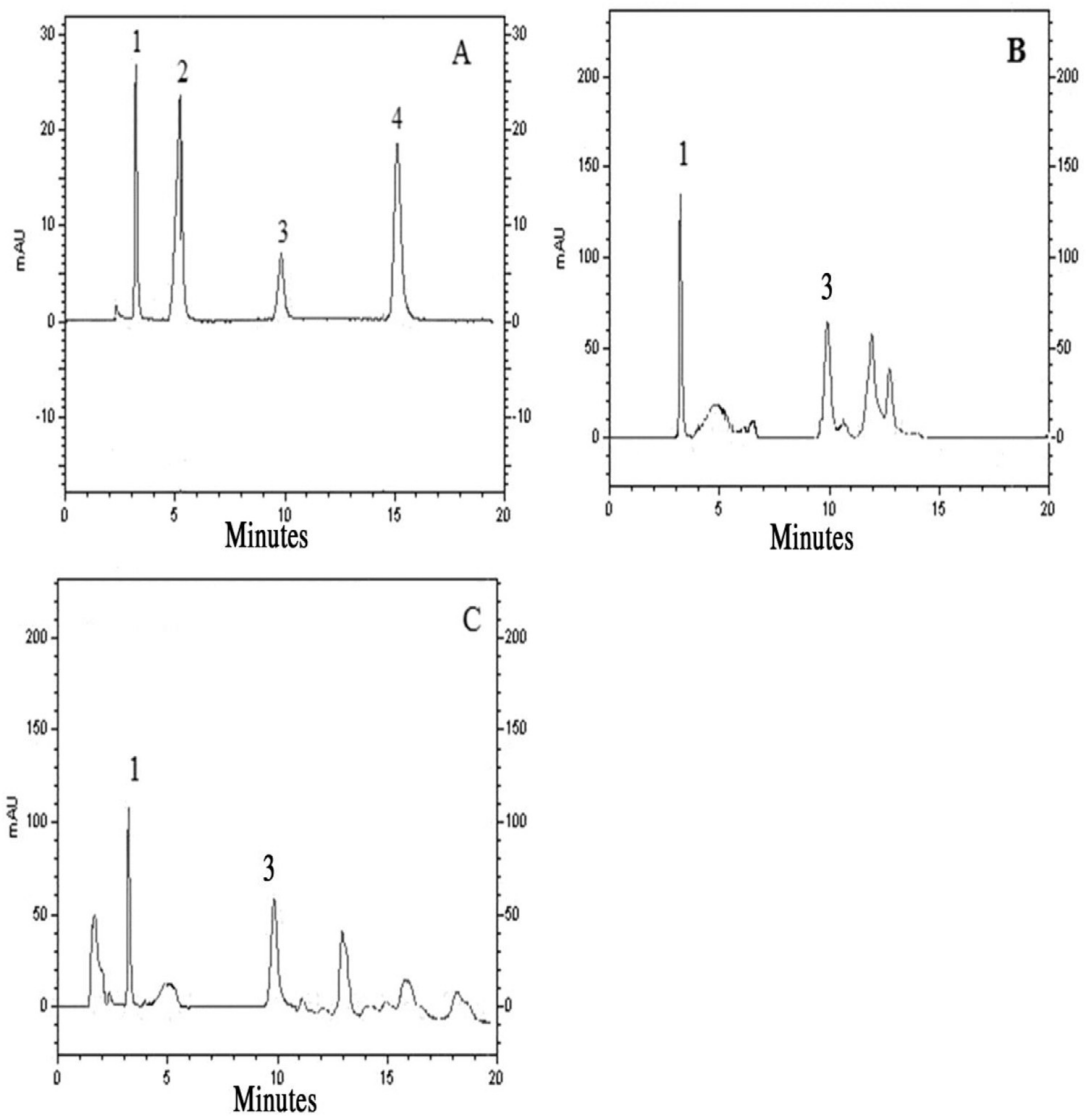
The chromatogram of (A) standard of analytes (1; MTC), (2; FPC), (3; IBF), (4; CZP); (B) human urine; and (C) human serum, spiked at 100 μg  L^−1^of each drug, after MNPs based V- D- a μ-SPE method under optimal conditions.

HPLC-UV chromatograms of the human serum, urine, and standard solution were shown in Fig. 12. The peaks of MTC, FPC, IBF, and CZP have appeared at retention times 3.2, 5.5, 9.8, and 15.2 min, respectively with acceptable disconnection and without any interference. In the human serum and urine sample, MTC and IBF were recognized, respectively. In the wastewater sample, none of the species was observed.

### Comparison of V-D-μ-SPE with other reported methods

In Table 5, linear dynamic ranges (LDRs), limits of detection (LODs), limits of quantification (LOQs), correlation of determination (r2), the relative standard deviation (RSD) and extraction recoveries (ER %), were compared with Micro UHPLC-MS/MS with on-line SPE system [2], montmorillonite-Ca modified carbon paste electrode [7], SPE using multi-template molecularly imprinted polymer [8], chromatography electron ionization mass spectrometry (GC-EI MS) [57], and spectrophotometric and fluorimetric [18], to extraction MTC, FPC, IBF, and CZP were reported. Based on the findings of the figures of merit, it seems that our method has shown similar or better results.

**Table 5:** Comparison of MNPs based V-D-μ-SPE method with reported methods for the determination of MTC, FPC, IBF, and CZP.

Method	LDR	LOD	LOQ	*R*^2^	*R* (%)	RSD (%)	Ref
Micro UHPLC-MS/MS	0.31–21.7 μg L^−1^	0.25 μg L^−1^	0.5 μg L^−1^	0.9950	–-	<5.2	
Electrochemical	3 × 10^−6^–1 × 10^−8^ mol L^−1^	3× 10^−9^ mol L^−1^	1× 10^−8^ mol L^−1^	0.998	>99.4	1.5	
SPE using multi-template molecularly imprinted polymer	—	1 μg L^−1^	3.33 μg L^−1^	0.999	>97	4.2	
GC-EI MS	2.5–10 μg L^−1^	0.58 μg L^−1^	1.75 μg L^−1^	0.9987		2.4–3.7	
Spectrophotometric and fluorimetric	0.316–3.16 μg L^−1^	0.13 μg L^−1^	–-	0.98	<94.4	5.42	
MNPs based V-D- μ-SPE	0.1–500 μg L^−1^	0.62–0.32 μg L^−1^	0.25–1.08 μg L^−1^	>0.9951	>94.3	<5.2	This work

## Conclusions

In this study, for the first time, a novel method of simultaneous extraction, determination, and pre-concentration of the acidic and basic drugs (MTC, FPC, IBF, and CZP) from human serum, urine and wastewater samples by using synthesized Fe_3_O_4_@SiO_2_@Kit-6@NH_2_ NPs based V-D-μ-SPE method was developed and validated. By using Mini-Tab software and response surface method (RSM) based on PBD, the first parameters were screened and then by using Box-Behnken design, the experiments were optimized and designed. The proposed method is simple, fast, and selective. The measurement of the mentioned drugs was accomplished without any interferes of ascorbic acid and aspirin. This method was used to determine drugs in serum, urine, and wastewater that showed good selectivity of adsorbent, low LODs, repeatability, reproducibility, and suitable rate recoveries in comparison to other proposed methods.

## Declaration of Competing Interest

The authors confirm that there are no conflicts of interest.
